# Evaluating Non-ablative Erbium Yttrium Aluminium Garnet (YAG) Laser Treatment for Polypropylene Mesh-Induced Vaginal Erosion: A Case Series

**DOI:** 10.7759/cureus.55128

**Published:** 2024-02-28

**Authors:** Nobuo Okui, Yuko Kouno, Kaori Nakano, Machiko A Okui

**Affiliations:** 1 Dentistry, Kanagawa Dental University, Kanagawa, JPN; 2 Urology, Dr. Okui's Urogynecology and Urology, Yokosuka, JPN; 3 Urogynecology, Yokosuka Urogynecology and Urology Clinic, Kanagawa, JPN

**Keywords:** vaginal erosion, laparoscopic sacrocolpopexy, transvaginal mesh, polypropylene mesh, pelvic organ prolapse, non-ablative erbium: yttrium-aluminum-garnet laser treatment

## Abstract

Background

Vaginal erosion caused by the polypropylene mesh is a serious side effect, and the development of effective treatment methods is required. This study explored the potential of non-ablative vaginal erbium yttrium aluminum garnet (YAG) laser treatment (VEL) as a novel treatment approach.

Methods

In this study, VEL was performed on nine women who experienced vaginal erosion after undergoing treatment for pelvic organ prolapse (POP) with polypropylene mesh. These patients visited our hospital between April and December 2020. Using the Renovalase (SP Dynamis Fotona d.o.o., Ljubljana, Slovenia), the laser was applied to the entire vagina, with intensive irradiation focused on the erosion areas. Detailed analyses of symptoms before and after treatment, as well as histopathological changes, were conducted one year post-treatment.

Results

Nine women were referred to our hospital due to vaginal erosion caused by polypropylene mesh. The participants' average age was 73.2 years (range: 69-81 years), with four patients having undergone transvaginal mesh (TVM) surgery and five undergoing laparoscopic sacrocolpopexy (LSC). The average time from mesh insertion to treatment initiation was 7.2 years (range: 3-15 years), with eight patients having previously attempted mesh removal. Post-treatment, significant improvements were observed in managing vaginal erosion and related bleeding, corroborated by histopathological analysis confirming cell regeneration and tissue repair. These improvements also resulted in significant improvements in bleeding management and quality of life (QoL).

Conclusion

VEL suggests the possibility of being an effective treatment method for vaginal erosion caused by a polypropylene mesh. However, further research is needed because of the small sample size and the limitations inherent in the retrospective case series design.

## Introduction

The use of polypropylene mesh for the treatment of pelvic organ prolapse (POP) is widely recognized as an effective solution. However, this approach carries the risk of serious complications such as vaginal erosion, bleeding, and damage to adjacent organs, which can profoundly impact patients' quality of life (QoL) [1, 2]. Furthermore, the incidence of mesh-related complications can vary depending on the amount and method of mesh usage, underscoring the importance of understanding how these factors influence the risk of reoperation and mesh erosion [3]. Complications from the use of synthetic meshes, including infections and inflammation, also present significant considerations in the selection of treatment strategies [4]. Moreover, in vitro characterization of mesh-induced soft tissue erosion can aid in understanding the physical interactions that lead to complications [5]. Recent studies on the assessment and treatment of POP highlight the need for a comprehensive evaluation of treatment approaches [6]. Vaginal erosion caused by polypropylene mesh is known to induce severe symptoms, such as pain and bleeding, and managing these complications poses a significant challenge for clinicians [7]. Traditionally, mesh removal has been considered a treatment method for vaginal erosion caused by mesh; however, this is associated with considerable risks, and an effective treatment method has yet to be developed.

This study examined whether non-ablative vaginal erbium yttrium aluminum garnet (YAG) laser treatment (VEL) could serve as an effective treatment option for vaginal erosion caused by polypropylene mesh. The removal of mesh for pain after mid-urethral sling surgery and the potential effectiveness of VEL in managing pain and tissue recovery have been reported [8]. The exploration of combination therapy with VEL and a neodymium laser as an effective treatment option for vulvodynia is ongoing. VEL has shown potential in effectively stopping bleeding and promoting the regeneration of cells around areas of vaginal erosion [9]. This study aimed to demonstrate the potential of laser treatment as a new approach to treating vaginal erosion caused by mesh. Through a case series of nine patients, it provided a detailed analysis of histopathological changes before and after laser treatment, exploring how laser treatment can effectively manage vaginal erosion and related bleeding.

## Materials and methods

Ethical approval and informed consent

In this retrospective case series study, the effectiveness of VEL in treating vaginal erosion caused by a polypropylene mesh was assessed. The study protocol received approval from the Ethics Committee of Yokosuka Urogynecology and Urology Clinic. All participants in this study provided written informed consent, explicitly agreeing to the review of their medical records for research purposes before receiving any treatment.

Study design and data collection

This retrospective analysis involved reviewing the medical records of women treated at our clinic for mesh-related vaginal erosion between April and December 2020. We collected data on patient demographics, clinical presentations, details of the VEL treatments administered, and post-treatment outcomes. The aim was to assess the therapeutic effectiveness and safety profile of VEL.

Selection and exclusion criteria

Eligible participants were women who experienced vaginal erosion following the use of polypropylene mesh for POP treatment within the study period. The inclusion criteria were as follows: women aged ≥20 years who presented with mesh-related vaginal erosion and for whom other treatment modalities had failed or were deemed insufficient. Exclusion criteria included patients undergoing antiplatelet or anticoagulant therapy, those with immunocompromised conditions, individuals with serious comorbid chronic diseases, pregnant or breastfeeding patients, and those with known allergies or hypersensitivity to laser treatment.

Laser treatment protocol

**Figure 1:**
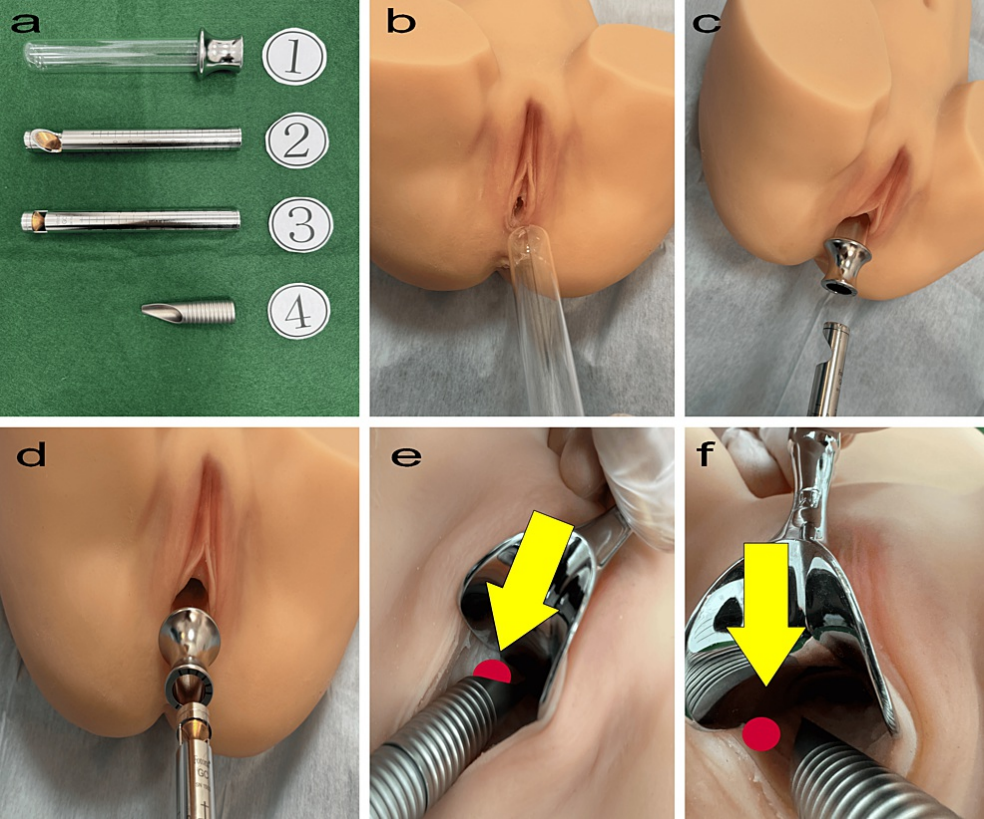
VEL treatment procedure. a. SP Dynamis, Copyright © 2013. Courtesy of Fotona d.o.o. (Ljubljana, Slovenia). This image in Figure 1a is provided by Fotona d.o.o. free of charge: 1, Special glass speculum for laser treatment; 2, PS03-GA angular adapter laser probe for the anterior vaginal wall; 3, R11-GC circular adapter laser probe for treating the entire circumference of the vagina; 4, PS03 adapter probe for direct erosion irradiation. b. Insertion of a glass speculum. c. Laser irradiation of the posterior vaginal wall using the PS03-GA. d. Whole-vaginal laser irradiation with the R11-GC. e. Targeted irradiation of erosions (indicated by the yellow arrow and red circle) using the PS03. f. An additional aspect of the procedure, as shown in e. The model was developed by Nobuo Okui. VEL: Vaginal erbium yttrium aluminum garnet (YAG) laser treatment.

Figure 1 illustrates the laser procedure, with photographs highlighting erosion on the posterior vaginal wall. The lesion, measuring 1.5 cm in size, is marked with a red circle in the images to clearly indicate its location and extent. Patients received local anesthesia with 8% xylocaine spray (Sandoz KK, Tokyo, Japan) for 15 minutes, followed by laser irradiation using RenovaLase (SP Dynamis Fotona d.o.o., Ljubljana, Slovenia). Various handpieces connected to the SP Dynamis laser were utilized. The VEL steps involved inserting a glass speculum into the vagina, using the PS03 handpiece with specific settings to irradiate the eroded areas, comprehensive vaginal irradiation with the R11 handpiece, and finally, targeted treatment of erosions with the PS03 handpiece.

Evaluation methods

Clinical Evaluation

The degree of bleeding due to vaginal erosion before and after treatment was recorded and assessed using the visual analog scale (VAS), which ranged from 0 (no bleeding) to 10 (worst imaginable bleeding).

*Quality-of-Life (QoL) Evaluation* 

Changes in quality of life before and after treatment were evaluated by the patients using the VAS. To address the multifaceted nature of QoL, this evaluation implicitly incorporates assessments of physical health (including pain), psychological health (such as anxiety and depression), social relationships (including issues related to odors), and sexual function. We chose to employ the VAS as it provides a holistic measure, allowing patients to reflect on their overall well-being across these dimensions in an integrated manner, rather than relying on distinct scales for each aspect.

Histopathological Evaluation 

Tissue samples were collected before treatment and one year after laser treatment to evaluate the effects of laser treatment on tissue regeneration and repair under a microscope.

Evaluation time frame

The study adhered to a standardized treatment protocol for all participants. The initial two VAS scores were measured before treatment (T0). The first treatment was administered at T0, with the VAS score reassessed on the same day. One month after the first treatment (L2), the second treatment was administered, and all VAS scores were evaluated before laser irradiation during the L2 visits. One month after the second treatment (L3), a third treatment was administered, followed by all VAS evaluations. All VAS scores were reassessed one month after the L3 treatment (T1) and finally, one year after the beginning of treatment (T12) at the final follow-up evaluation. The aim was to assess the impact of this treatment protocol on patients' bleeding and quality of life (using VAS scores) over time. Trained medical personnel consistently conducted all VAS score measurements to accurately assess the effects of treatment.

Statistical analysis

Data were analyzed using Python (Python Software Foundation, Wilmington, DE, USA) with the assistance of ChatGPT (OpenAI, San Francisco, CA, USA). Differences in clinical evaluations, histopathological evaluations, and quality of life assessments before and after treatment were evaluated using paired t-tests, with p-values < 0.05 considered statistically significant.

## Results

Patient characteristics

During the specified period, nine female patients were referred to our facility due to non-improving erosions caused by polypropylene mesh. All patients expressed a desire for laser treatment. Table 1 outlines the characteristics of the nine patients. The average age of the participants was 73.2 years (range: 69-81 years), with four undergoing transvaginal mesh (TVM) surgery and five undergoing laparoscopic sacrocolpopexy (LSC) surgery. The average duration from mesh insertion to treatment was 7.2 years (range: 3-15 years), with eight patients having previously attempted mesh removal without improvement in their erosions. One patient, referred to as Case f, did not attempt mesh removal due to embarrassment over vaginal odor, which hindered her from seeking consultation with a surgeon.

**Table 1:** Patient characteristics. LSC: Laparoscopic sacrocolpopexy; TVM: Transvaginal mesh; TVT: Tension-free vaginal tape; LET: Local estrogen therapy.

No.	Age (Years)	BMI (kg/m^2^)	Polypropylene Mesh Insertion Method and Timing	Hysterectomy Performed	Location of Mesh Erosion	Mesh Erosion Total Size (cm) and Number of Locations	Duration of Erosion (Years)	Previous Treatments	Medical History
a	69	24.5	LSC, 6 years ago	Yes	Anterior and posterior vaginal wall	3 cm (2 locations)	5	Partial mesh removal	None
b	73	25.6	LSC, 4 years ago	No (uterus preserved)	Anterior vaginal wall	4 cm (2 locations)	2	Partial mesh removal	Diabetes
c	80	30	TVM, 12 years ago with concurrent TVT	No (uterus preserved)	Anterior and posterior vaginal wall	3 cm (3 locations)	8	Partial mesh removal	Hypertension, Diabetes
d	73	17.2	TVM, 15 years ago (post-hysterectomy)	Yes (after hysterectomy for uterine fibroids)	Anterior vaginal wall	2.5 cm (1 location)	10	Partial mesh removal, LET	Hypertension
e	69	28.1	LSC, 4 years ago	Yes	Anterior vaginal wall	2 cm (1 location)	3	Partial mesh removal	None
f	74	23.3	LSC, 4 years ago	Yes	Vaginal apex	2.5 cm (2 locations)	4	No treatment	Hypertension,
g	81	32	TVM, 15 years ago with concurrent TVT	Yes (post-hysterectomy for uterine prolapse)	Vaginal apex	2.5 cm (1 location)	10	Partial mesh removal, LET	Hypertension
h	70	27.8	TVM, 10 years ago	Yes	Anterior and posterior vaginal wall	3 cm (2 locations)	8	Partial mesh removal	None
i	69	21.4	LSC, 3 years ago	Yes	Vaginal apex	1.5 cm (1 location)	3	Partial mesh removal	None

Pre-treatment pathological analysis

**Figure 2:**
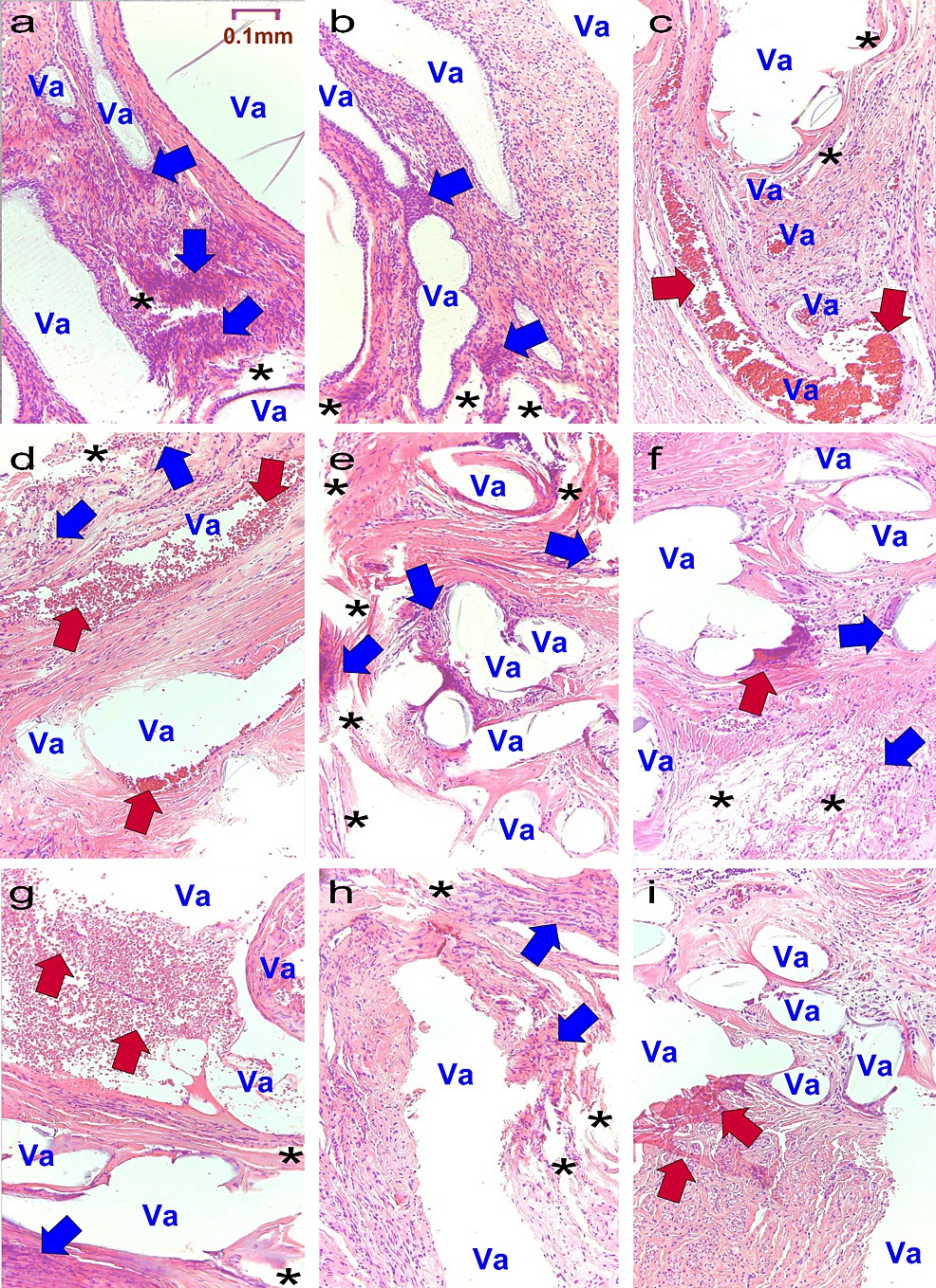
Pre-treatment biopsy tissue. a: Case a; b: Case b; c: Case c; d: Case d; e: Case e; f: Case f; g: Case g; h: Case h; i: Case i. Va: Vacuoles formed when mesh fibers dislodged during the preparation of pathological specimens. Blue arrows: Foreign-body giant cells. Red arrows: Sites of bleeding. *: Cracks in the surrounding tissue caused by the fraying of polypropylene mesh fibers due to aging. All images were stained with H&E and captured at an optical magnification of 40x using an Olympus System Microscope BX43 and objective lens LPLN10X (Olympus Corporation, Tokyo, Japan).

Figure 2 displays the biopsy tissues before treatment in all cases.

In case a, the site of mesh insertion formed vacuoles due to polypropylene mesh fibers falling out during the preparation of the microscopic specimen (Va). The fibers are frayed due to aging, causing destruction of the surrounding tissue and resulting in large vacuoles. Additionally, foreign-body giant cells form aggregates and abscesses (blue arrow). Healthy tissue is affected by fine damage from fibers, leading to cracks (*).

In case b, multiple deformed vacuoles were observed, similar to case a, along with enlarged cracks in the tissue (*).

In case c, adjacent to the enlarged vacuoles (Va), a large hematoma was observed (red arrow), with fine cracks (*).

In case d, the vacuoles were deformed (Va), accompanied by a hematoma (red arrow).

In case e, it is presumed that the fiber size was relatively maintained, with vacuoles approximately the same size as the fibers being prominent (Va). However, some vacuoles ruptured, starting abscess formation (blue arrow).

In case f, due to varying conditions of the fibers, the vacuole sizes are inconsistent (Va), with minor bleeding present (red arrow).

In case g, the vacuoles could not maintain their shape (Va) and formed a large hematoma (red arrow).

In case h, vacuoles collapsed and merged with adjacent vacuoles (Va), with cracks observed throughout the tissue.

In case i, the vacuoles have healed or changed due to the disruption of fibers (Va), with bleeding observed (red arrow).

Clinical evaluation analysis

**Figure 3:**
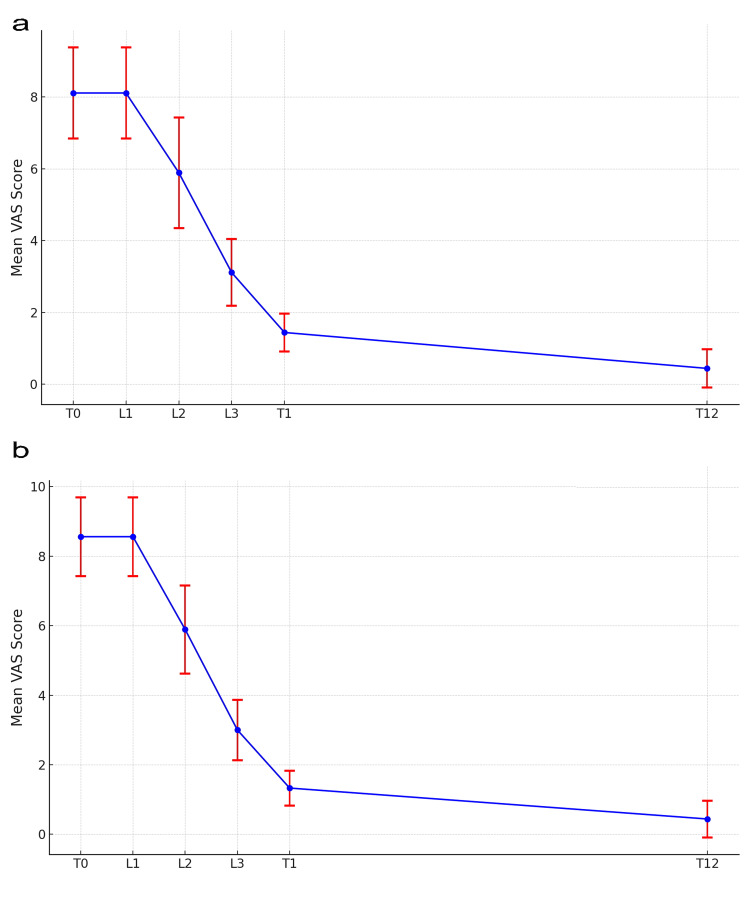
Temporal changes in bleeding and QoL. a: VAS evaluation of bleeding. Vertical axis: VAS score; horizontal axis: time. b: VAS evaluation of QoL. Vertical axis: VAS score; horizontal axis: time. VAS: Visual Analogue Scale; QoL: Quality of Life. T0: At initial consultation; L1: After the first laser treatment; L2: After the second laser treatment; L3: After the third laser treatment; T1: One month after L3; T12: 12 months after L3.

Figure 3a depicts the bleeding from erosions assessed using VAS. The average VAS score at T0 was 8.11 ± 1.27, with L1 showing no change from T0. The average VAS at L2 was 5.89 ± 1.54; at L3, it was 3.11 ± 0.93; at T1, it was 1.44 ± 0.53; and at T12, it was 0.44 ± 0.53. The change from T0 to T12 was statistically significant (p < 0.001).

Figure 3b illustrates the evaluation of QoL using the VAS. The average VAS score at T0 was 8.56 ± 1.13. At L1, the score remained similar to T0. By L2, the average VAS decreased to 5.89 ± 1.27, indicating that the treatment significantly reduced bleeding over a short period. The average VAS score further decreased to 3.00 ± 0.87 at L3, and by T1, the score was reduced to an average of 1.33 ± 0.50, significantly improving the patient's QoL. At the T12 follow-up, the VAS score decreased to an average of 0.44 ± 0.53, revealing the long-term effects of the treatment. The change from T0 to T12 was statistically significant (p < 0.001).

**Figure 4:**
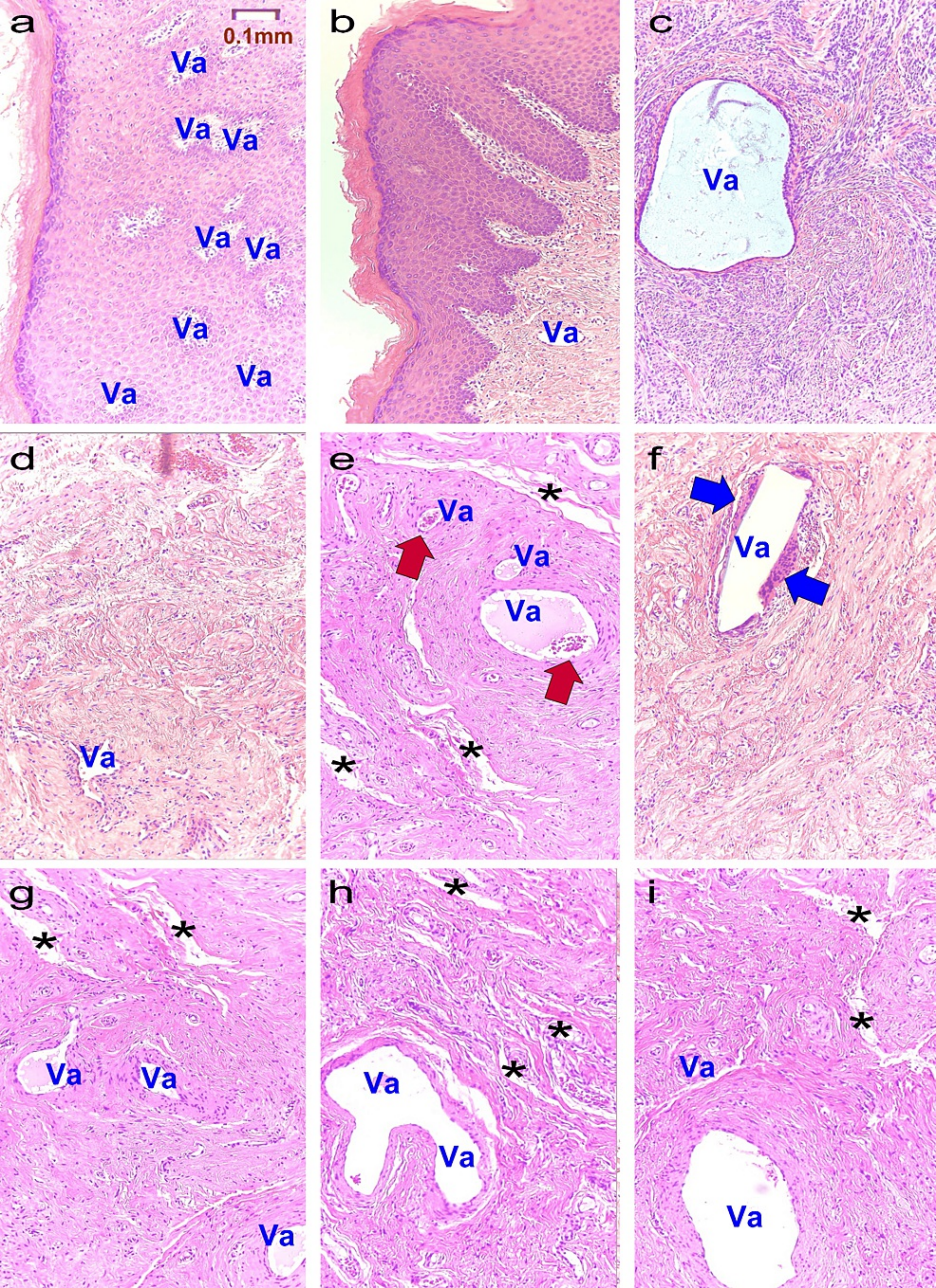
Post-treatment pathological analysis. a: Case a; b: Case b; c: Case c; d: Case d; e: Case e; f: Case f; g: Case g; h: Case h; i: Case i. Va: Vacuole formed when the mesh fell off during pathological specimen preparation. Blue arrows: Foreign-body giant cells. Red arrows: Sites of bleeding. *: Cracks in the surrounding tissue caused by the fraying of polypropylene mesh fibers due to aging.

Figure 4 presents histological images of the lesion sites at T12. In all cases, cell proliferation has filled the previously fiber-induced tissue deficits, with fibers remaining and forming minimal-sized vacuoles (Va). In case a, numerous small vacuoles are observed. In case b, significant epithelial regeneration is noted, with fibers being moved deeper into the tissue. In case c, enlarged vacuoles are visible due to the disintegration of fibers, surrounded by cells. Case d exhibits only one vacuole. Meanwhile, cases e and f show minimal rebleeding. In cases e, g, and h, it is apparent that cells, once densely regenerated, are now being split by parts of frayed fibers (*). In cases, e, f, h, and i, similar to case c, large vacuoles are present due to deformed fibers but are surrounded by cells.

 

No side effects were associated with VEL treatment. There were no cases of cystitis, urethritis, or vaginitis, and there was no increase in temporary pain.

## Discussion

In this study, the effectiveness of VEL as a treatment option for vaginal erosion caused by polypropylene mesh was investigated. The main findings indicate a significant improvement in vaginal erosion after VEL treatment, with a reduction in patient symptoms and enhancement of QoL. These outcomes suggest that VEL treatment is a viable option for managing vaginal erosion.

First, examining the study subjects, Pacquée S et al. reported mesh erosion in 6 out of 30 women treated for POP with Prolift polypropylene mesh for the anterior and/or posterior vaginal wall [1]. Chughtai B et al.'s study showed an association between the amount of vaginal mesh used for POP and SUI repair and the risk of mesh erosion and reoperation, with the combination of mesh and sling showing the highest risk of erosion and reintervention [2]. In our study, erosions were observed on the anterior or posterior vaginal walls. Additionally, tension-free vaginal tape (TVT) sling surgeries were performed on two patients. Notably, eight patients attempted mesh removal, which was unsuccessful for all, implying that removing the mesh does not necessarily prevent the remnant mesh from protruding through the erosion.

Second, considering total mesh removal, Shi C et al. reported a 2.70% rate of post-TVM surgery pain with a 38% mesh exposure rate in the pain group [10]. Schimpf MO et al. reported that mesh erosion occurred in up to 36% of patients, although reoperation rates were low [3]. This suggests many patients currently suffer from bleeding and pain without undergoing surgery, despite mesh erosion. Van Rest KL et al. argued that the duration of symptoms should not deter surgery if a correlation between symptoms and mesh location is observed [11]. However, Kasyan G et al. reported that mesh-related complications included erosion (4.8%), vaginal adhesions (0.3%), mesh protrusion into the bladder (0.15%), vesicovaginal fistula, mesh protrusion (0.3%), mesh contraction (1%), and dyspareunia and pain (2.4% of cases) [7]. Therefore, complete mesh removal can be challenging and risky, potentially leading to various complications. Furthermore, Padoa A et al. analyzed complications and difficult pain treatment after mesh removal and reported that approximately one-third of women with pain complications showed improvement in symptoms even after complete mesh removal [12]. Our study indicated that all nine patients showed potential for bladder or rectal damage from mesh removal. Nonetheless, with significant worsening of bleeding and QoL, any alternative treatment to removal was deemed detrimental to further patient observation.

Third, pretreatment pathology is considered. Pathological examination of the erosive area at T0 showed characteristic vacuole formation. Wang H et al. elucidated that polypropylene mesh, frayed and peeled due to aging, elicits a significant inflammatory response in the surrounding tissue [13]. They reported an increase in the number of M2 subtype macrophages and T lymphocytes in severely degraded mesh, with a trend of increasing along with mesh degradation [13]. Taylor D et al.'s study characterized soft tissue erosion by surgical mesh in vitro, focusing on a significant postoperative complication known as "mesh erosion," which occurs when mesh materials wear down adjacent soft tissues through friction, reporting erosion rates consistent with clinical experience [5]. Our study found that the longer the mesh was inserted, the more the fibers degraded, showing abrasion and cracks around them. In addition, vacuoles along the mesh shape merged and enlarged, with some forming abscesses and hematomas.

Fourth, the post-VEL pathological findings are discussed. Gaspar A et al. reported changes in the vaginal mucosa after VEL [14]. Post-VEL treatment, the thickness of the vaginal mucosa increased from 45 µm (10-106 µm) to 153 µm (97-244 µm), with significant cell proliferation observed [14]. Previous research has reported that even in cases with severe vaginal pain, the average thickness of the vaginal wall before treatment was 57.3±14.2 µm, whereas post-treatment it was 133.3±24.5 µm [8]. Our study is distinctive in the pathology of cell proliferation surrounding the vacuoles, as reported by Wang H et al. and Taylor D et al., with an overall increase in cell numbers. However, some patients showed rebleeding on pathological examination, suggesting the need for additional VEL treatments every one to two years.

Finally, clinical findings and persistence were considered. The improvement in QoL, as measured by the VAS, is noteworthy and associated with hysterectomy. According to Erel CT et al., the effect of VEL treatment on SUI in women post-hysterectomy is equivalent to that in women who have not undergone hysterectomy, but the beneficial effect of VEL treatment on SUI may be limited in duration in women who have undergone hysterectomy [15]. A previous study reported a duration of 1.5 months for VEL treatment in patients with interstitial cystitis [16]. Gambacciani M et al. reported that VEL for the genitourinary syndrome of menopause lasts about two years [17]. These findings suggest that VEL, which improves bleeding and pain without mesh removal, requires repetition of VEL treatment every one to two years from a pathological perspective.

The limitations of this study include the small sample size and retrospective case series design, limiting generalization and causation estimation. The ambiguity of long-term treatment effects and the lack of direct comparison with other treatments should also be considered when interpreting the results.

## Conclusions

This study demonstrated the potential of VEL as an effective therapy for vaginal erosion caused by polypropylene mesh. Conducted on nine women suffering from vaginal erosion, the treatment explored how VEL could effectively manage vaginal erosion and associated bleeding through a detailed analysis of symptoms and histopathological changes before and after treatment. Pre-treatment pathology revealed tissue destruction and inflammatory responses due to the polypropylene mesh, while post-treatment findings confirmed significant regeneration of cells and repair of tissues. This was also reflected in notable improvements in bleeding and QoL. This approach introduces a new possibility for the treatment of vaginal erosion caused by polypropylene mesh, suggesting the potential use of VEL to promote the regeneration of cells around vaginal erosions and effectively stop bleeding.

However, this study has limitations, including a small sample size and a retrospective case series design, necessitating caution in generalizing the results and estimating causality. Future research, including the verification of the long-term effects of treatment and direct comparisons with other therapeutic methods, is anticipated to further expand and deepen these initial findings.
